# *Dryopteris* *filix-mas *(L.) Schott ethanolic leaf extract and fractions exhibited profound anti-inflammatory activity

**Published:** 2019

**Authors:** Earnest Oghenesuvwe Erhirhie, Chika Ndubuisi Emeghebo, Emmanuel Emeka Ilodigwe, Daniel Lotanna Ajaghaku, Blessing Ogechukwu Umeokoli, Peter Maduabuchi Eze, Kenneth Gerald Ngwoke, Festus Basden Gerald Chiedu Okoye

**Affiliations:** 1 *Department of Pharmacology and Toxicology, Faculty of Pharmaceutical Sciences, Nnamdi Azikiwe University, Awka, Nigeria.*; 2 *Department of Pharmacology, Faculty of Pharmaceutical Sciences, Enugu State University of Science and Technology, Enugu State, Nigeria.*; 3 *Department of Pharmaceutical and Medicinal Chemistry Faculty of Pharmaceutical Sciences, Nnamdi Azikiwe University, Awka, Nigeria.*; 4 *Department of Pharmaceutical Biology and Biotechnology, Faculty of Pharmaceutical Sciences, Nnamdi Azikiwe University, Awka, Nigeria.*

**Keywords:** Dryopteris filix-mas, Anti-inflammatory, Rheumatoid arthritis, Quercetin 3-O-α-L-rhamnopyranoside, Non-ulcerogenic

## Abstract

**Objective:**

*Dryopteris filix-mas *(*D. filix-*mas) (L.) Schott, (Dryopteridaceae) is used in traditional medicine, particularly in the Southern parts of Nigeria for the treatment of inflammation, rheumatoid arthritis, wounds and ulcers. In this study, we evaluated the anti-inflammatory activity of its ethanolic leaf extract and fractions.

**Materials and Methods:**

The ethanolic leaf extract and fractions were screened for anti-inflammatory properties using egg-albumin-induced paw edema, xylene-induced topical ear edema, formaldehyde-induced arthritis and ulcerogenic models. The ethyl acetate most promising vacuum liquid chromatography fraction (VLC-E7) was purified using size exclusion chromatography technique (Sephadex LH-20) and its structure was elucidated using nuclear magnetic resonance (NMR) and mass spectrometry. Total phenolic and flavonoid contents were also determined.

**Results:**

From the study, ethyl acetate and butanol fractions elicited better anti-inflammatory activities in egg-albumin-induced paw edema, formaldehyde-induced arthritis and xylene-induced topical ear edema. The ethanol extract, ethyl acetate and butanol fractions were non-ulcerogenic at 200 and 400 mg/kg. The compound isolated from Sephadex fraction (SPH-E6) was quercetin-3-O-α-L-rhamnopyranoside.

**Conclusion:**

Results of this study justify the ethnomedicinal use of *D. filix-mas *leaf for treatment of inflammation and rheumatoid arthritis. We suggest that *D. filix-mas* could be a prospective anti-inflammatory agent with no gastric irritation side effect, due to its bioactive component, quercetin-3-O-α-L-rhamnopyranoside.

## Introduction

Inflammation is an adaptive physiological response of the body to foreign agents such as toxic chemicals and allergens, as well as infections, burns and other stimuli that could cause tissue injuries (Alam et al., 2013 ▶; Shrestha et al., 2014 ▶). Unregulated level of inflammation is associated with several disorders, including rheumatoid arthritis, inflammatory bowel diseases, cancer, diabetes, and cardiovascular diseases (Murugesan and Deviponnuswamy, 2014 ▶).

Most conventional non-steroidal anti-inflammatory drugs (NSAIDs) have not proven satisfactory outcomes, but are rather accompanied by adverse effects such as gastric irritation, peptic ulcer, hepatotoxicity, nephrotoxicity, hypertension, etc. (Ezeja et al., 2015 ▶).

Due to the limitations posed by conventional NSAIDs in the management of inflammatory disorders, it is imperative to explore alternative agents from natural sources, particularly medicinal plants which are used by the majority of the world population, as over 80% rely on traditional medicines for various health disorders (Yadav and Agarwala, 2011 ▶; Hajhashemi et al., 2012 ▶).

Medicinal plants, the richest bio-resources of alternative medicine are widely applicable in the management of various pathological conditions due to their accessibility, acceptability, affordability and high-safety profile (Erhirhie et al., 2018 ▶).

*Dryopteris filix-mas *(Dryopteridaceae), commonly known as male fern, dryopteris or water loving fern, is an evergreen plant growing up to 60-150 cm. It is found in stream, moist environments, open grounds, stone and brick walls (Uwumarongie, 2016 ▶). Its leaf decoction is popularly used by traditional healers in various parts of Edo and Delta States, Nigeria as a therapy for inflammation, rheumatoid arthritis, ulcers and wounds (Personal communication). Its reported pharmacological activities include antioxidant and cytotoxic (Sekendar et al., 2012 ▶), antimicrobial (Soare et al., 2012 ▶), antihelmintic (Urban et al., 2014), antidiarrheal (Uwumarongie, 2016 ▶) and tocolytic (Bafor et al., 2017 ▶) activities.

Considering the traditional benefits of *D. filix-mas *leaf in curbing inflammatory disorders among Southern Nigerian populace, this study evaluated its anti-inflammatory properties and also characterized its bioactive anti-inflammatory component using bioassay- guided purification and isolation approaches.

## Materials and Methods

**Instruments, apparatus, chemicals and reagents**

Analytical high pressure liquid chromatography (HPLC) Agilent 1100 series, HPLC-mass spectrometer (Agilent), visible spectrophotometer (721G, Zhejiang Top Cloud-Agri Technology, China), separating funnel (Pyrex, England), xylene and acetic acid (BDH, England), ethanol, methanol, ethyl acetate, butanol and dichloromethane (JHD, China), Sephadex LH-20 (GE Healthcare Bio-science AB, Sweden), Folin-Ciocalteu reagent (Loba Chemie, India).

**Experimental animals**

Albino rats and mice of both sexes were procured from the animal facility of the Faculty of Veterinary medicine, University of Nigeria, Nsukka, Nigeria. Animals were handled in compliance with the National Institute of Health Guidelines for the care and use of laboratory animals (Pub. No. 85-23, revised 1985) as approved by the Faculty of Pharmaceutical Sciences, Nnamdi Azikiwe University, Nigeria.

**Plant collection and authentication**

Fresh leaves of *D. filix-mas *were collected from a swampy site in Amawbia, Awka South Local Government Area, Anambra State, Nigeria. The plant specimen was authenticated by Dr. Akinnibosun H.A., a plant taxonomist in the Department of Plant Biology and Biotechnology, Faculty of Life Science, University of Benin, Nigeria. It was deposited in the herbarium of the Department and a voucher number, UBH_d_285A was allocated to it. 

**Plant extraction, fractionation, isolation and structural elucidation**

Fresh leaves of *D. filix-mas *were washed and dried at room temperature for 7 days. A total of 2.5 kg of pulverized leaves was extracted by maceration using 7.5 liters of 80% aqueous ethanol for a period of 48 hr. Filtrate recovered was concentrated to dryness using a water bath at 40^o^C (Azubike et al., 2015 ▶). The extract was partitioned successively against n-hexane, ethyl acetate, butanol and water (Ajaghaku et al., 2017 ▶). The various fractions obtained were concentrated to dryness using thermostatic water bath at 40^o^C. Thereafter, 6.5 g of the most active fraction (i.e. ethyl acetate fraction) was subjected to vacuum liquid chromatography (VLC) using 500 mL of various ratios of n-hexane: ethyl acetate mixtures (10:0, 9:1, 8:2, 7:3, 6:4, 5:5, 4:6, 3:7, 2:8, 1:9 and 0:10) and dichloromethane (DCM): methanol mixtures (10:0, 9:1, 7:3, 5:5, 2:8, and 0:10) which resulted in 17 pooled fractions (Ajaghaku et al., 2017 ▶). The ethyl acetate VLC fraction (VLC-E7, N: E, 0:10), selected based on its promising anti-inflammatory activity, was subjected to size exclusion chromatography using Sephadex LH-20 and eluted using dichloromethane and methanol (1:1). Analytical HPLC was carried out by a Dionex P580 HPLC system coupled with a photodiode array detector (UVD340S) using methanol and nanopure water as the mobile phase. Detection was done at 235, 254, 280 and 354 nm. Nuclear magnetic resonance (NMR) spectra (1H) were recorded by Bruker ARX 500 NMR. Mass spectrometric electron spray ionization (MS-ESI) was obtained by Finnigan LCQ Deca mass spectrometer (Ajaghaku et al., 2017 ▶).

**Phytochemical test**

The extract and fractions were screened for the presence of secondary metabolites using methods described by Yadav and Agarwala (2011) ▶.

**Estimation of total phenolic and** **flavonoid** **contents**

The amount of phenol and flavonoid in the extract and fractions were determined using Folin-Ciocalteu reagent and aluminium chloride methods, respectively as described by Yadav and Agarwala (2011). Total phenolic contents are expressed as gallic acid equivalent (GAE mg/g). Total flavonoid contents are presented in mg quercetin equivalent per g dry weight (mg QE/g). 

**Acute toxicity study**

This assessment was carried out using the method of Miller and Tainter as described by Randhawa (2009) ▶. Two species of animals (60 albino mice ( 23.47±0.35 g) and 60 albino rats ( 129.86±0.98 g) of both sexes were fasted overnight, divided into six groups of ten animals each as follows; one control group, and five groups treated with different doses (100, 1000, 2000, 3000 and 5000 mg/kg) of the extract. After administration, special attention was given during the first 4 hours and after 24 hr for signs of toxicity or death. Animals were further observed for two weeks for signs of delayed toxicity and death.

**Anti-inflammatory assays**

**Egg-albumin-induce paw edema test**

This test was done using the methods described by Anosike and Obidoa (2010). Sixty albino rats of both sexes (134.93±0.89 g) were randomly allocated into twelve groups of five animals each as follow: Control, indomethacin (received 10 mg/kg), extract (received 200 and 400 mg/kg), n-hexane fraction (received 200 and 400 mg/kg), ethyl acetate fraction (received 200 and 400 mg/kg), butanol fraction (received 200 and 400 mg/kg) and water fraction (received 200 and 400 mg/kg). One hour after administration of different treatments, initial paw diameter (0 hr) was measured with the aid of a cotton thread wrapped around the perimeter of the hind paw. Thereafter, 0.1 ml of fresh egg-albumin was injected into the sub-planter region of the left hind paw of animals and subsequent paw diameters were measured and recorded at 1^st^, 2^nd^, 3^rd^, 4^th ^and 5^th^ hour consecutively after the injection of egg-albumin. Paw edema was estimated as the difference between the paw diameter at zero hour (Vo) and the paw diameter at other time intervals (Vt) after the administration of the fresh egg-albumin. Percentage inhibition of paw edema in test groups was calculated relative to paw edema in control group at various time points. Doses of extract and fractions were selected based on the preliminarily determined median effective dose (ED_50_).

**Effects on formaldehyde-induced arthritis**

From the outcome of the egg-albumin induced paw edema test, ethyl acetate, butanol, water fractions and extract were selected for formalin-induced arthritis as described by Nworu et al (2012) ▶. Fifty albino rats (118.06±0.81 g) of both sexes were randomized into 10 groups of five animals each, as follows: Group 1 (control, received distilled water 10 mL/kg), group 2 (received indomethacin 5 mg/kg), groups 3-10 (received 200 and 400 mg/kg doses of either the extract, ethyl acetate, butanol or water fractions). One hour after treatment, paw diameter was measured and arthritis was induced by injection of 0.1 mL of 2.5% formaldehyde solution on the sub-plantar region of the left hind paw. On the 4^th^ day of the experiment, formalin injection was repeated for the maintenance of arthritis. Arthritis level was assessed by measuring the paw diameter once daily for 10 days. The level of inhibition of arthritis in test groups relative to the control group was calculated using “area under curve” approach using Microsoft Excel, 2010.

**Effects of the extract and fractions on xylene-induced topical edema**

This test was carried out using the method described by Ajaghaku et al (2013) ▶. A total of sixty five albino mice (23.25±0.34 g) of both sexes, were randomized into thirteen groups of five mice each, as follows: Group 1 (control, received methanol 50 µL/ear), group 2-3 (received indomethacin 50 and 100 µg/ear), groups 4-13 (received 50 and 100 µg/ear of extract, n-hexane, ethyl acetate, butanol and water fractions). After grouping, 50 µL of various treatments were applied on the anterior surface of the mice right ear, while 50 µL of xylene was applied on the posterior surface of the same ear. After two hours, mice were sacrificed by cervical dislocation and both right and left ears were removed with the aid of 4-mm diameter cork borer and weighed using analytical weighing balance. Edema was quantified as the weight difference between right and left ear plug of animals. Percentage inhibition of edema in test groups was calculated relative to edema of the control group.

The ethyl acetate fraction and its selected VLC fractions, namely VLC-E13, VLC-E5, VLC-E14, and VLC-E7, were also screened using the same procedure. A total of sixty albino mice (23.32±0.26 g) of both sexes, were randomized into 12 groups of 5 mice each. Indomethacin served as positive control.

Sephadex fractions were likewise tested. Sixty five albino mice (28.22±0.36 g) of both sexes, were randomized into 13 groups (n=5). The groups included control, indomethacin, VLCE-7, SPH-E5, SPH- E6, SPH- E3, and SPH-E4. Each sample had two dose levels (50 and 100 µg/ear).

**Ulcerogenic test**

This test was conducted using the method described by Moke et al (2015) ▶. Forty albino mice (20.02±0.44 g) of both sexes, were fasted for 18 hr before the experiment and randomized into eight groups of 5 animals each and were treated as follows: control (received distilled water 10 mL/kg), indomethacin (50 mg/kg), and 200 and 400 mg/kg dose of either extract, butanol or ethyl acetate fractions. Four hours after administration, animals were sacrificed by cervical dislocation and their stomachs were removed, cut open along the grater curvature, washed with tap water and observed for the presence or absence of lesions. Lesions on mucosal surface were scored using the following arbitrary scale: 0=no lesion (normal stomach); 0.5=hyperemia; 1=one or two lesions (spot ulcer); 2=hemorrhagic streak, 3=deep ulcer, and 4=perforation.

**Data analysis**

Results are presented as mean±standard error of mean (SEM) of sample replicates. Significant differences between control and treatment groups were determined by one-way analysis of variance (ANOVA) followed by *post-hoc* Turkey’s test. A p<0.05 was considered statistically significant. Statistical Package for Social Science (SPSS- version-20.0 for Windows, Inc., Chicago, IL, USA) was used for data analysis.

## Results

**Acute toxicity**

There were no signs of toxicity or death following treatment with various doses of the extract (up to 5000 mg/kg) 24-hours after treatment of rats and mice. No toxicity and death were found after the 14-day observation period. 

**Phytochemical constituents**

Tannins, flavonoids, saponins, steroids, alkaloids, terpenoids and reducing sugar were present in the leaf extract and fractions of *D. filix-mas*. Cardiac glycoside was absent in the butanol fraction (Table 1).

**Table 1:** Qualitative phytochemistry results

	**Extract**	**n-hexane fraction**	**Ethyl-acetate fraction**	**Butanol fraction **	**Water fraction**
**Tannins**	++	++	++	+++	++
**Flavonoids**	+++	+	++	++	++
**Saponins**	++	+	+	++	+
**Steroids**	++	++	++	++	++
**Alkaloids**	++	+	+	+	+
**Terpenoids**	++	+	+	+	++
**Anthraquinolones**	-	-	-	-	-
**Cardiac glycosides**	+	+	+	-	+
**Reducing sugar**	+	+	+	+	+

“-”: absent, “+”: trace, “++”: moderate and “+++”: abundant.

Ethyl acetate fraction (34.93±0.04 mg GAE/g) revealed the highest total phenolic content. This was followed by butanol fraction (31.35±0.07 mg GAE/g), extract (21.06±0.14 mg GAE/g) and n-hexane fraction (15.78±0.11 mg GAE/g) (Table 2). 

Total flavonoid content among samples tested was in the following order water fraction (12.50±0.83 mg QE/g) < butanol fraction (40.83±1.67 mg QE/g) < extract (44.17±1.67 mg QE/g) < n-hexane fraction (54.17±0.83 mg QE/g) < ethyl acetate fraction (107.50±0.83 mg QE/g) (Table 2).

**Table 2:** Total flavonoid and phenolic content of extract and fractions

	**Total phenolic content (mg GAE/g)**	**Total flavonoid content (mg QE/g)**
**Extract**	21.06 ± 0.14	44.17 ± 1.67
**n-hexane fraction**	15.78 ± 0.11	54.17 ± 0.83
**Ethyl-acetate fraction**	34.93 ± 0.04	107.50 ± 0.83
**Butanol fraction**	31.35 ± 0.07	40.83 ± 1.67
**Water fraction**	19.40 ± 0.04	12.50 ± 0.83

Values represent mean±SEM of duplicates. GAE: Gallic acid equivalent. QE: Quercetin equivalent.

**Anti-inflammatory results**

**Effects on egg-albumin-induced paw edema**

Administration of 200 and 400 mg/kg doses of extract and fractions caused a reduction in paw edema when compared to control group. Ethyl acetate and butanol fractions elicited a dose-dependent inhibition against egg-albumin-induced paw edema that was more marked than other groups. Their activities were more significant at the 3^rd^ and 4^th^ hour (Table 3).

**Effects on formaldehyde-induced arthritis**

The extract, ethyl acetate and butanol fractions, but not water fraction, produced significant reductions (p<0.05) in AUC when compared to the control group. Indomethacin had the highest inhibition against arthritis compared to other treatments (Table 4).

**Table 3:** Effect of extract and fractions on egg-albumin induced paw edema.

		Paw edema (cm) and inhibition in paw edema (%)				
	mg/kg	1^st^ hour	2^nd^ hour	3^rd^ hour	4^th^ hour	5^th^ hour
Control:	-	0.78 ± 0.05	0.80 ± 0.04	0.80 ± 0.04	0.75 ± 0.04	0.67 ± 0.05
Indomethacin	10	0.72 ± 0.06 (8%)	0.64 ± 0.07 (20%)	0.57 ± 0.06* (29%)	0.49 ± 0.04* (35%)	0.51 ± 0.07 (24%)
Extract	200	0.70 ± 0.05 (10%)	0.64 ± 0.05 (20%)	0.63 ± 0.06* (21%)	0.57 ± 0.05* (24%)	0.52 ± 0.05 (22%)
	400	0.64 ± 0.04 (18%)	0.62 ± 0.01* (23%)	0.59 ± 0.01* (26%)	0.56 ± 0.02* (25%)	0.48 ± 0.03* (28%)
NHF	200	0.79 ± 0.03 (0%)	0.78 ± 0.04 (2%)	0.73 ± 0.03 (9%)	0.70 ± 0.03 (7%)	0.68 ± 0.03 (0%)
	400	0.73 ± 0.02 (6%)	0.71 ± 0.03 (11%)	0.72 ± 0.04 (10%)	0.70 ± 0.03 (7%)	0.64 ± 0.02 (5%)
EAF	200	0.70 ± 0.03 (10%)	0.65 ± 0.02 (19%)	0.59 ± 0.01* (26%)	0.58 ± 0.03* (23%)	0.52 ± 0.02 (23%)
	400	0.57 ± 0.05* (27%)	0.57 ± 0.03* (29%)	0.56 ± 0.03* (30%)	0.50 ± 0.03* (33%)	0.50 ± 0.03 (25%)
BF	200	0.59 ± 0.02* (24%)	0.59 ± 0.03* (26%)	0.55 ± 0.02* (31%)	0.53 ± 0.01* (29%)	0.50 ± 0.02 (25%)
	400	0.58 ± 0.03* (26%)	0.54 ± 0.01* (33%)	0.53 ± 0.03* (34%)	0.48 ± 0.02 (36%)	0.44 ± 0.02* (34%)
WF	200	0.75 ± 0.03 (4%)	0.72 ± 0.03 (10%)	0.68 ± 0.03 (15%)	0.66 ± 0.02 (12%)	0.61 ± 0.02 (9%)
	400	0.70 ± 0.03 (10%)	0.71 ± 0.02 (11%)	0.65 ± 0.02 (19%)	0.63 ± 0.04 (16%)	0.57 ± 0.05 (15%)

Values are presented as mean±Standard error of mean (SEM) of sample replicates (n=5).

* P<0.05: Significantly different from control group. NHF: n-hexane fraction, EAF: Ethyl acetate fraction, BF: Butanol fraction, and WF: Water fraction.

**Table 4:** Effect of extract and fractions on formaldehyde-induced arthritis

	Dose	AUC	Percentage inhibition (%)
Control:	-	7.24±0.11	
Indomethacin	5 mg/kg	4.65 ± 0.12*	35.77
Extract	200 mg/kg	6.09±0.15*	15.88
	400 mg/kg	6.03±0.32*	16.77
Ethyl acetate fraction	200 mg/kg	5.86±0.19*	19.12
	400 mg/kg	5.35±0.17*	26.08
Butanol fraction	200 mg/kg	5.25±0.25*	27.51
	400 mg/kg	4.95±0.16*	31.63
Water fraction	200 mg/kg	7.11±0.12	1.80
	400 mg/kg	6.82±0.08	5.86

Values are presented as mean±Standard error of mean (SEM) of sample replicates (n=5).

* p<0.05: Significantly different from control group. AUC: Area under curve.

**Effect of extract and fractions on xylene-induced ear edema**

The extract and fractions exhibited a strong inhibition of topical inflammation induced by xylene on the mice ears. These inhibitions were significant (p<0.05) when compared to the control group. When compared with the standard control, better activities were observed following treatment with the extract, and ethyl acetate and butanol fractions. Among the fractions, ethyl acetate fraction exerted the highest activity while n-hexane fraction produced the least activity (Table 5). 

**Effects of** **selected VLC fractions on xylene-induced ear edema**

All selected VLC fractions showed more than 50% inhibition against xylene-induced topical ear edema in mice (Table 6).

**Table 5:** Effect of extract and fractions on xylene-induced ear edema

		Weight of ear (mg)			
	Dose (µg/ear)	Left ear	Right ear	Edema (mg)	Percentage Inhibition (%)
Control:	-	4.16 ± 0.04	6.54 ± 0.05	2.38 ± 0.04	
Indomethacin	50	4.04 ± 0.08	5.00 ± 0.07	0.96 ± 0.06*	59.66
	100	4.10 ± 0.03	4.86 ± 0.05	0.76 ± 0.02*	68.07
Extract	50	3.72 ± 0.02	4.60 ± 0.03	0.88 ± 0.02*	63.03
	100	3.94 ± 0.07	4.58 ± 0.04	0.66 ± 0.05*	72.27
N-hexane fraction	50	3.30 ± 0.05	5.34 ± 0.05	2.04 ± 0.09	14.29
	100	3.48 ± 0.06	5.40 ± 0.03	1.92 ± 0.04	19.33
Ethyl acetate fraction	50	4.06 ± 0.05	4.80 ± 0.05	0.74 ± 0.04*	68.91
	100	4.06 ± 0.02	4.56 ± 0.05	0.50 ±0.04*	78.99
Butanol fraction	50	3.96 ± 0.09	4.64 ± 0.05	0.68 ± 0.06*	71.43
	100	4.00 ± 0.03	4.62 ± 0.04	0.62 ± 0.04*	73.95
Water fraction	50	3.32 ± 0.02	4.58 ± 0.06	1.26 ± 0.06	47.06
	100	3.56 ± 0.05	4.58 ± 0.04	1.02 ± 0.04	57.14

Values are presented as mean ± Standard error of mean (SEM), n=5.

* p<0.05: Significantly different from control.

**Effects of selected Sephadex fractions on xylene-induced ear edema**

Sephadex fractions inhibited xylene-induced topical ear edema in mice when compared with the control group. All tested samples produced inhibition above 50%, except 50 µg of SPH-E4 (Table 7).

**Table 6:** Effect of selected VLC fractions on xylene-induced ear edema

		Weight of ear (mg)			
	Dose/ear	Left Ear	Right Ear	Difference (mg)	Percentage Inhibition (%)
Control: Xylene	50 µl	4.72 ± 0.15	8.76 ± 0.64	4.04 ± 0.54	
Indomethacin	50 µg	5.50 ± 0.05	7.08 ± 0.11	1.58 ± 0.16*	60.89
	100 µg	5.06 ± 0.07	6.38 ± 0.16	1.32 ± 0.11*	67.33
VLC-E13	50 µg	5.53 ± 0.02	6.95 ± 0.05	1.43 ± 0.07*	64.70
	100 µg	4.86 ± 0.28	5.70 ± 0.32	0.84 ± 0.25*	79.21
VLC-E5	50 µg	4.60 ± 0.10	5.40 ± 0.14	0.80 ± 0.05*	80.20
	100 µg	5.20 ± 0.16	6.50 ± 0.54	1.30 ± 0.39*	67.82
VLC-E14	50 µg	4.98 ± 0.07	6.88 ± 0.39	1.90 ± 0.45*	52.97
	100 µg	4.82 ± 0.12	5.42 ± 0.07	0.60 ± 0.08*	85.15
VLC-E7	50 µg	5.20 ± 0.29	6.36 ± 0.20	1.16 ± 0.37*	71.29
	100 µg	4.82 ± 0.04	5.82 ± 0.19	1.00 ± 0.19*	75.25
Ethyl acetate fraction	50 µg	4.88 ± 0.06	6.50 ± 0.06	1.62 ± 0.04*	59.90
	100 µg	4.62 ± 0.20	5.98 ± 0.23	1.36 ± 0.14*	66.34

Values are presented as mean ± Standard error of mean (SEM), n=5.

* p<0.05: Significantly different from control. VLC: Vacuum liquid chromatography. EAF: Ethyl acetate fraction.

**Table 7:** Effect of selected Sephadex fractions on xylene-induced ear edema

		Weight of ear (mg)				
	Dose/ear	Left Ear	Right Ear	Difference (mg)		Percentage Inhibition (%)
Control:	50 µl	4.62±0.25	9.90±0.45	5.28±0.24		
Indomethacin	50 µg	4.26±0.05	6.50±0.07	2.24±0.09*	57.58	
	100 µg	4.46±0.05	5.82±0.21	1.36±0.17*	74.24	
VLCE-7	50 µg	4.38±0.10	6.96±0.12	2.58±0.06*	51.14	
	100 µg	4.30±0.08	5.78±0.22	1.48±0.19*	71.97	
SPH- E4	50 µg	4.46±0.05	7.26±0.18	2.80±0.14*	46.97	
	100 µg	4.30±0.14	6.26±0.19	1.96±0.07*	62.88	
SPH-E5	50 µg	4.24±0.08	6.70±0.11	2.46±0.16*	53.41	
	100 µg	4.46±0.09	6.08±0.11	1.62±0.12*	69.32	
SPH- E6	50 µg	4.02±0.13	6.32±0.13	2.30±0.03*	56.44	
	100 µg	4.22±0.09	5.82±0.13	1.60±0.14*	69.70	
SPH- E3	50 µg	3.98±0.12	6.38±0.21	2.40±0.14*	54.55	
	100 µg	4.08±0.11	5.76±0.25	1.68±0.19*	68.18	

Values are presented as mean±Standard error of mean (SEM), n=5.

* p<0.05: Significantly different from control. SPH: Sephadex.

**Effects on gastric mucosa integrity**

There were spot ulcers in indomethacin-treated group. However, administration of the extract, ethyl acetate and butanol fractions did not produce gastric lesions (Table 8).

**Table 8:** Effect of extract and fractions on gastric mucosa

**Treatment **	**Dose (mg/kg)**	**Ulcer score**
**Control**	-	0.00 ±0.00
**Indomethacin**	50	1.00±0.00
**Extract**	200	0.00±0.00
	400	0.00±0.00
**Butanol fraction**	200	0.00±0.00
	400	0.00±0.00
**Ethyl acetate**	200	0.00±0.00
	400	0.00±0.00

Values are presented as mean ± Standard error of mean (SEM), n= 5.

**HPLC chromatogram and UV spectrals of major compounds detected in extract and selected fractions**

**Figure 1:**
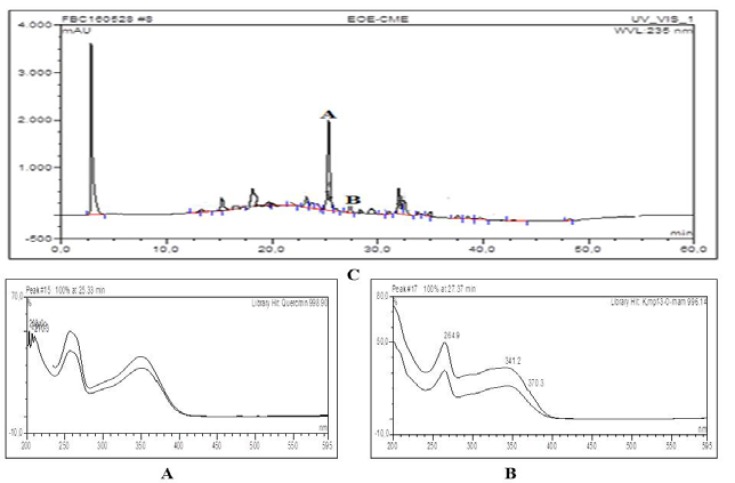
HPLC chromatogram and UV spectrals of major compounds detected in the ethanolic leaf extract of *D. filix mas*. A= Quercitrin (Rt=25.33 min, 998.90) and B=Kmpf-3-O-rham (Rt=27.37, 996.14)

**Figure 2:**
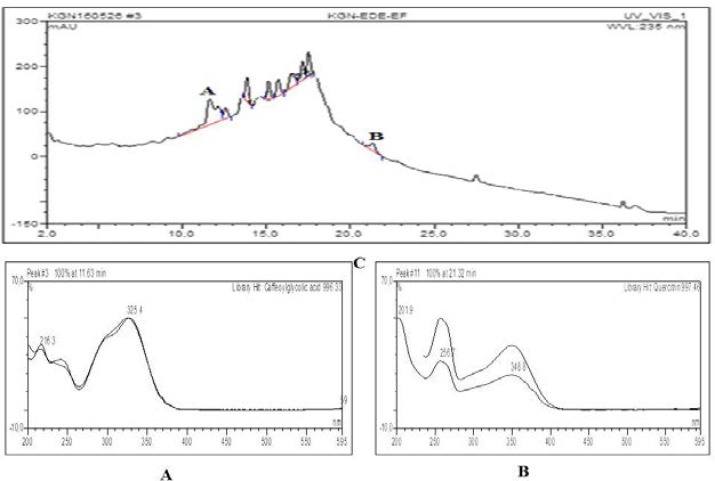
HPLC chromatogram and UV spectrals of major compounds detected in ethyl acetate fraction of *D. filix mas* A=Caffeoylglycolic acid (Rt=11.63 min, 996.33) and B=Quercitrin (Rt=21.32 min, 997.46)

**Figure 3:**
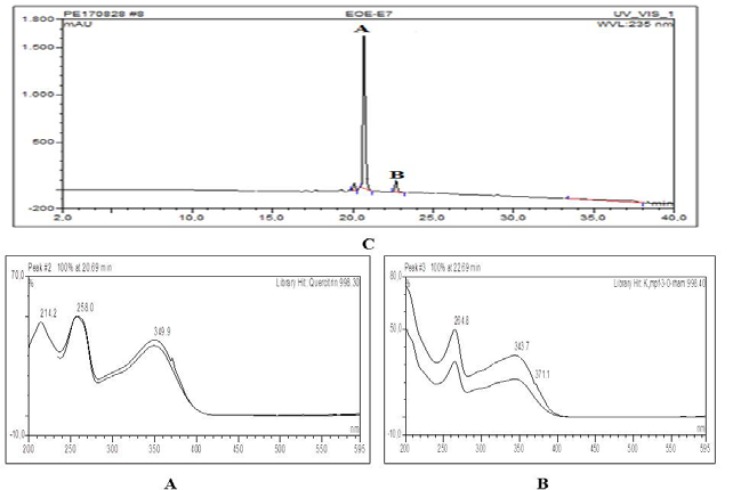
HPLC chromatogram and UV spectrals of major compounds detected in VLC-E7 fraction. A=Quercitrin (Rt=20.69 min, 998.30) and B=Kmpf-3-O-rham (Rt=22.69 min, 998.40)

**Figure 4:**
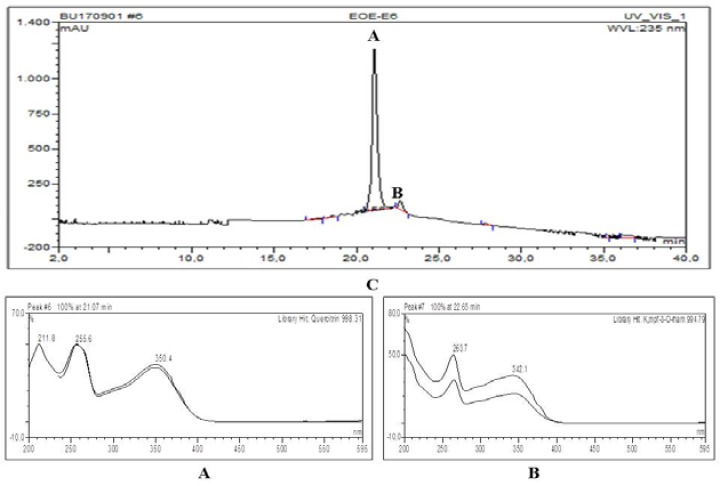
HPLC chromatogram and UV spectrals of major compounds detected in in SPH-E6 fraction. A= Quercitrin (Rt=21.07 min, 998.31) and B= Kmpf-3-O-rahm (Rt=22.65 min, 994.79)

Figures 1–4 show quercitrin as a major compound in extract and fractions.

**Quercetin-3O-α-L-rhamnopyranoside isolated from** ***D.*** ***filix-mas***

**Figure 5:**
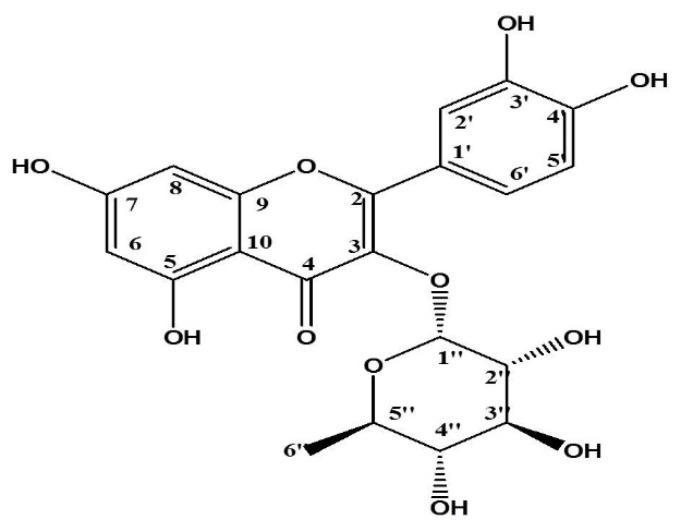
Structure of isolated compound, Quercetin-3O-α-L-rhamnopyranoside

This pure amorphous yellow powder was isolated from Sephadex LH-20 column chromatography using absolute MeOH as eluent. MALDI-TOF-MS: m/z 449[M+H]+, 471[M+Na]+; UV λ_max _nm (MeOH): 256.8 and 350.4; ^1^H-NMR (600MHz, MeOH, δ): 0.94 (3H, d, *J*=6.0 Hz, H_3_-6’’), 3.40 (1H, d, H-5’’), 3.54 (1H, m, H-4’’), 3.75 dd (1H, *J*=9.2, 3.4 Hz, H-3’’), 4.22 (1H, dd, *J*=3.4, 1.7 Hz, H-2’’), 5.35 (1H, d, *J*=1.7 Hz, H-1’’), 6.21 (1H,d, *J*=2.1 Hz, H-6), 6.38 (1H, d, *J*=2.1 Hz, H-8), 6.91 (1H,d, *J*=8.2 Hz, H-5’), 7.31 dd (1H, dd, *J*=8.2, 2.1 Hz, H-6’), and 7.34 d (1H, d, *J*=2.0 Hz, H-2’) (Table 9 and Figure 5).

**Table 9:** H1 NMR data of isolated compound, quercetin-3O-α-L-rhamnopyranoside compared with literatures

No	Δh	(δH in Acetone), Ghaly et al. (2010)	(δH in MeOH), Tae-Seong et al., (2015)
1	-		
2	-		
3	-		
4	-		
5	-		
6	6.21 d (J=2.1 Hz, 1H)	6.22(1H,d, J=2.3Hz, H-6)	6.20(1H,d,J=2Hz,H-6)
7	-		
8	6.38 d (J=2.1 Hz, 1H)	6.43(1H, d, J=2.3Hz, H-8)	6.36(1H,d,J=2.0,H-8)
9	-		
10	-		
1’	-		
2’	7.34 d (J=2.0 Hz, 1H)	7.46 (J=2.0, d, 1H, H-2’)	7.34 (J=2.2,1H,d,H-2’)
3’	-		
4’	-		
5’	6.91 d (J=8.2 Hz, 1H)	6.95 (1H,d, H=8.4Hz, H-5’)	6.9 (1H,d,J=8.5,Hz, H-5’)
6’	7.31 dd (J=8.2, 2.1 Hz, 1H)	7.34(1H,dd,J=8.4,2.3Hz,H-6’	7.31(J=2.2Hz, 8.5Hz,1H,dd, H-6’)
1’’	5.35 d (J=1.7 Hz, 1H)	5.45(1H,d, J=1.5Hz, H-1’’)	5.36 (1H,D,J=8.5Hz, H-1’’
2’’	4.22 dd (J=3.4, 1.7 Hz, 1H)	3.30-3.32 (4H,H2’’-H5’’)	4.23(1H,dd,,J=1.63Hz,H-2’’)
3’’	3.75 dd (J=9.2, 3.4 Hz, 1H)	3.30-3.32 (4H,H2’’-H5’’)	3.76(1H,dd,J=3.4,3.23Hz,H-3’’
4’’	3.54 (m 1H)	3.30-3.32 (4H,H2’’-H5’’)	3.66(1H,m,H-4’’)
5’’	3.4 (d 1H)	3.30-3.32 (4H,H2’’-H5’’)	3.42(1H,m,H-5’’)
6’’	0.94 d (J=6.0 Hz, 3H)	0.88(3H,d, J=5.6Hz, Me-6’’)	0.95(3H,d,J=6.14Hz,H-6’’

## Discussion

Inflammatory disorders constitute a major global health challenge. Although synthetic drugs for the alleviation of these disorders are dominating the market, their adverse effects raise a lot of concerns (Minaiyan et al., 2018 ▶; Ruckmani et al., 2018 ▶). 

Safety and efficacy govern scientific exploration of natural products as alternative anti-inflammatory agents. The present research therefore evaluated the anti-inflammatory activity of *D. filix –mas, *a traditional herbal remedy used mainly among Southern Nigerian populace against inflammation and rheumatoid arthritis. 

In the current study, acute toxicity test revealed the absence of toxic symptoms or mortality at 5000 mg/kg dose, suggesting that *D. filix-mas* leaf extract is safe in the treatment of acute inflammatory conditions.

From the anti-inflammatory results, significant reductions in paw edema by the extract, ethyl acetate and butanol fractions of *D. filix-mas*, especially at the 3^rd^ and 4^th^ hour, suggest their ability to inhibit acute phase of inflammatory responses which is characterized by the release of inflammatory mediators such as histamine and serotonin which are associated with the first phase as well as prostaglandin and bradykinin which are associated with the second phase. Suppression of rats paw edema may be attributed to the phytoconstituents such as flavonoids and alkaloids which are present in the extract and fractions. The secondary metabolites of these plants have been reported to elicit anti-inflammatory properties (Okigbo et al., 2009 ▶; Yadav and Agarwala, 2011 ▶). Qnais et al. (2014) ▶ reported that the extract of *Artemisia herba-alba* exhibited anti-inflammatory properties in a rat paw edema model due to the presence of flavonoids. A study done by Ilavarasan et al. (2005) ▶ revealed that the extract of *Cassia fistula *elicited anti-inflammatory activity in paw edema model due to the presence of flavonoids. Alkaloids from several medicinal plants were also reported to reduce rat and mouse paw edema induced by egg-white and carrageenan (José et al., 2006 ▶).

The phlogistic agent, xylene is a commonly used chemical in the assessment of acute inflammation. Application of xylene is known to cause irritation of the living tissues, increase in prostaglandin E_2_ production, increase in fluid accumulation and edema production at the site of inflammation (Okoye et al., 2014 ▶). From the results of this study, inhibition of xylene-induced ear edema by the extract and fractions suggests that they possess activity against acute inflammation and could also be useful in the management of skin-related inflammatory disorders. Presence of flavonoids and tannins in the extract and fractions of *D filix-mas *may have contributed to the reduction in topical edema. Tannins were reported to exert anti-inflammatory activities in topical edema induced by various phlogistic agents (Mei and Jong, 2012 ▶). Flavonoids were also found to ameliorate ear edema and chronic skin inflammation (Jin et al., 2010 ▶). 

Appreciable anti-inflammatory activities elicited by the extract, ethyl acetate and butanol fractions of *D. filix-mas *in xylene-induced topical ear edema, prompted further evaluation of their possibilities to modulate chronic inflammatory condition using formalin, which was reported to mimic human arthritis through the release of inflammatory mediators such as prostaglandin (Nworu et al., 2012 ▶; Ajaghaku et al., 2013 ▶). From the results of formaldehyde-induced arthritis, administration of extract, ethyl acetate and butanol fractions caused significant reduction in AUC. This suggests that *D filix-mas *could be of relevance in the management of chronic inflammatory conditions such as rheumatoid arthritis. Alkaloids such as colchicine have been reported to be of use in the management of rheumatoid arthritis due to their abilities to reduce pains and swelling in chronic inflammatory conditions (José et al., 2016 ▶). Several alkaloids and flavonoids from medicinal plants have been reported to ameliorate arthritis induced by formaldehyde and other agents (José et al., 2016 ▶). Therefore, the presence of alkaloids and flavonoids in the extract and fractions of *D. filix-mas *may have contributed to its anti-arthritic activity.

Prolonged administration of NSAIDs is commonly associated with gastrointestinal bleeding and peptic ulcer (Chatterjee et al., 2014 ▶). Based on our findings, there were no gastric lesions in groups treated with the extract and fractions compared to indomethacin, a reference NSAID. This suggests that the extract and fractions of *D. filix-mas *are not associated with gastrointestinal irritation and could be a better choice in the management of chronic inflammatory disorders than conventional NSAIDs. A study done by Gretzer et al. (2001) ▶ revealed that damage to the gastric mucosa of a normal stomach can only occur when expressions of both COX-1 and COX-2 are inhibited. This may account for the absence of ulceration in gastric mucosa of animals that received the extract and fractions, suggesting that their anti-inflammatory mechanism is not directly associated with COX-1 inhibition but COX-2. To corroborate this, the flavonoids, quercitrin and kaempferol revealed by HPLC results of the extract and fractions of *D. filix-mas *have also been reported in other medicinal plants such as *Abrus cantoniensis, Bauhinia curvula Benth *to exert gastroprotective effects (Beber et al., 2018 ▶). 

Non-ulcerogenic activity of *D. filix-mas *may be due to the presence of alkaloids and flavonoids in its extract and fractions. The majority of flavonoids have been reported to inhibit COX-2 enzymes. In the same way, isoquercitrin, quercetin and kaempferol from other medicinal plants were shown to possess anti-COX activities (Díaz et al., 2016 ▶). 

The study also revealed quercitrin as the major compound detected in the extract and bioassay guided eluted fractions of *D. filix –mas.*

From the NMR results, presence of aglycon was very evident, giving the proton signals at δ-7.34, 7.31 and 6.91 assigned to the protons H-2’, H-6’ and H-5’, respectively. The two metacoupled doublet at δ 6.21 and 6.35 were also identical to the protons H-6 and H-8, respectively. Looking at the spectral data and compared to those reported by Ghaly et al. (2010) ▶ (δH in Acetone) and Tae-Seong et al. (2015) ▶ (δH in MeOH), the aglycon was identified as quercetin. The proton NMR showed the characteristics of α-L-rhamnopyranoside. Based on the proton NMR with the presence of an anomeric proton signal at δ5.35 and a methyl proton signal at δ0.95, our compound was confirmed to be quercetrin. The activities assigned to the flavonoid, quercetin-3O-α-L-rhamnopyranoside, which we identified in this work could confirm the traditional usefulness of *D. filix-mas *against inflammatory disorders.

From this study, we discovered that the extract and fractions of *D. filix-mas* elicited anti-inflammatory activities due to their bioactive flavonoid compound, quercetin-3O-α-L-rhamnopyranoside. Our study, therefore, validated the use of *D. filix-mas *against rheumatoid arthritis and inflammation.
